# Non-communicable diseases are key to further narrow gender gap in life expectancy in Shanghai, China

**DOI:** 10.1186/s12889-020-08932-x

**Published:** 2020-06-03

**Authors:** Hanyi Chen, Yi Zhou, Lianghong Sun, Yichen Chen, Xiaobin Qu, Hua Chen, Janani Rajbhandari-Thapa, Shaotan Xiao

**Affiliations:** 1 Department of Science Research and Information Management, Shanghai Pudong New Area Center for Disease Control and Prevention, Shanghai, 200136 China; 2 Fudan University Pudong Institute of Preventive Medicine, Shanghai, China; 3 College of Public Health, University of Georgia, Athens, GA, USA

**Keywords:** Life expectancy, Cause of death, Trends, Health equity, Mortality, Chronic disease, Joinpoint, Decomposition analysis, China

## Abstract

**Background:**

To address change in the gender gap of life expectancy (GGLE) in Shanghai from 1973 to 2018, and to identify the major causes of death and age groups associated with the change over time.

**Methods:**

The temporal trend in GGLE was evaluated using retrospective demographic analysis with Joinpoint regression. Causes of death were coded in accordance with the International Classification of Diseases and mapped with the Global Burden of Disease (GBD) cause list. The life table technique and decomposition method were used to express changes in GGLE.

**Results:**

The trend of GGLE in Shanghai experienced two phases, i.e., a decrease from 8.4 to 4.2 years in the descent phase (1973–1999) and a fluctuation between 4.0 and 4.9 years in the plateau phase (1999–2018). The reduced age-specific mortality rates tended to concentrate to a narrower age range, from age 0–9 and above 30 years in the descent phase to age above 55 years in the plateau phase. Gastroesophageal and liver cancer, communicable, chronic respiratory, and digestive diseases were once the major contributors to narrow GGLE in the descent phase. While, importance should be attached to a widening effect on GGLE by lung cancer, cardiovascular diseases, other neoplasms like colorectal and pancreatic cancer, and diabetes in the recent plateau phase.

**Conclusions:**

Non-communicable diseases (NCDs) have made GGLE enter a plateau phase from a descent phase in Shanghai, China. Public efforts to reduce excess mortalities for male NCDs, cancers, cardiovascular diseases, chronic respiratory diseases, and diabetes in particular and health policies focused on the middle-aged and elderly population might further narrow GGLE. This will also ensure improvements in health and health equity in Shanghai China.

## Background

The gender gap in life expectancy at birth (GGLE), which is the difference in life expectancy at birth (LE) by gender, was 4.4 years in 2016 (74.2 years for females and 69.8 years for males) [1]. There are gender differences in illnesses due to interactions among biological, social, psychological, and behavioral factors [2, 3], which may have led to gender differences in mortality, cause of death, and hence life expectancy at birth. While the gender ratio at birth in China is male-biased, the GGLE is female-biased and suggest a need for increased effort to understand the underlying cause of shorter male life expectancy at birth. Understanding long-term trends in GGLE and the cause of death will provide evidence for health professionals and policymakers to prioritize efforts and narrow gender inequity in LE.

Tremendous progress on LE has been found worldwide [4, 5], and recent studies find a narrowing GGLE in most high-income developed countries, for example, in countries like Sweden, France, and the UK [6–9]. China has also made great achievements in LE [10–12]. Among all its provinces, Shanghai has ranked first in LE in China mainland for many years [13], with low mortality even by the standards of high-income countries. Our previous study showed that after a period of decline and recovery, LE in Shanghai finally increased from 71 years in the 1970s to 84 years in 2018, and is still on the rise [14]. China has also made great strides in economic development. With economic development, the GGLE should continue decreasing as the gender gap is negatively associated with a nation’s per capita gross domestic product, meaning as countries develop the gender gap closes [15]. However, GGLE in China, more specifically in Shanghai-the economic hub of China, continues to persist. Shanghai has yet to see the GGLE inflection with male catching up with females’ LE.

To our knowledge, there has been no research on GGLE in Shanghai. Considering that China has attached great importance to LE and implemented “Healthy China 2030” program with aims to further improve national LE to 79 years by 2030, this study intends to address changes in GGLE in Shanghai from 1973 to 2018. This study aims to identify the major cause-of-death and age groups associated with narrowing or widening GGLE overtime. Moreover, the GGLE is both due to biological as well as behavioral factors [16]. Identifying the diseases associated with China’s GGLE and existent research on disease risk factors will inform Chinese policymakers on risky behaviors, particularly among men. This long-term trend study will also make up for the lack of research on GGLE trends across decades in mainland China.

## Methods

### Overview

We assessed GGLE trend in Shanghai local residents from 1973 to 2018, which traced back to the earliest time of full coverage of the Shanghai Mortality Registration System. We investigated different stages of the trend in GGLE and quantified the age- and cause-specific contributions to the change.

### Data sources

We used the 45-year mortality information from Shanghai Pudong New Area Center for Disease Control and Prevention (CDC). Individual data was anonymized and de-identified before analysis. Strict confidentiality of individual data was practiced during the entire study. Shanghai established a comprehensive Mortality Registration System in 1973, with full coverage of residents’ mortality information from medical institutions at all levels [17]. The system was further improved through routine verification with local population registry as well as funeral and cremation system and hence ensures the completeness of the registration system to the extent possible [18]. Details have been explained in our previous article [14]. The dataset in use is available on reasonable request.

### Coding and data quality

Rigorously trained clinicians coded cause of death. Historical data and data from 2002 were coded in accordance with the International Classification of Diseases, 10th Revision (ICD-10). Data from 1992 to 2001 were coded based on ICD-9. Moreover, historical records were checked against historical annual reports by cause and age to avoid inconsistency in classification.

According to the Global Burden of Disease (GBD) study, garbage codes should be redistributed to enhance the validity of public health analysis and are classified into four categories [19]. We redistributed garbage codes such as heart failure, peritonitis, and septicemia using the GBD recommended algorithms [19]. However, for senility and other unspecified cause of mortality lack medical records or causal inference information, we just kept the original ill-defined data. We evaluated the age-specific proportion of garbage codes and compared the data quality in different periods with those of other countries/regions. Finally, we mapped the 45-year data in accordance with the GBD cause list to make a comparison in the same framework.

### Causes of death

We focused our analysis on 14 mutually exclusive and exhaustive cause of death categories, which covered all the leading causes of death across the study years in Shanghai. Causes of death included the following: cardiovascular diseases (cerebrovascular diseases, ischemic heart diseases), neoplasms (lung cancer, stomach cancer, liver cancer, and esophageal cancer), chronic respiratory diseases, communicable diseases, digestive diseases, diabetes, endocrine, blood, and immune disorders, kidney diseases, and injuries. The causes of death we focused on and their corresponding ICD-9 and ICD-10 codes have been listed in Additional file 1: Table A1.

### Analysis

We applied Joinpoint regression to evaluate the temporal trend in GGLE during the 45 years of study period. The basic idea of the method is to model the time series using a few continuous linear segments [20]. Joinpoint regression tests whether a multi-segmented line is a significantly better fit than a straight or less-segmented line based on permutation tests [21]. In contrast, other regression methods investigate trends to find the best-fit line through years of data [22]. The application of Joinpoint method helps to find a meaningful turning point of a trend and avoids artificially dividing the long study period [23].

We calculated the gender-specific LE with the conventional life table technique based on annual abridged life tables. The age intervals of the life table were age 0–1, 1–4, 5–9, and in subsequent five-year age groups up to age 85 with an open end.

We used age- and cause-specific decomposition method to express changes of GGLE in years widened or narrowed at corresponding periods [24]. Narrowed GGLE can be attributed to (1) a faster drop in male mortality than female; (2) a slower rise in male mortality than female; (3) male mortality decreases while female’s increases. While an expanded GGLE is just the opposite. Details of the formulas can be found elsewhere [25].

Joinpoint regression analyses were carried out using the Joinpoint Regression Program, Version 4.0.4 (US National Cancer Institute, MD). Statistical analysis was performed with Stata/SE 13.0 (College Station, TX).

## Results

### Trend of GGLE

**Fig. 1:**
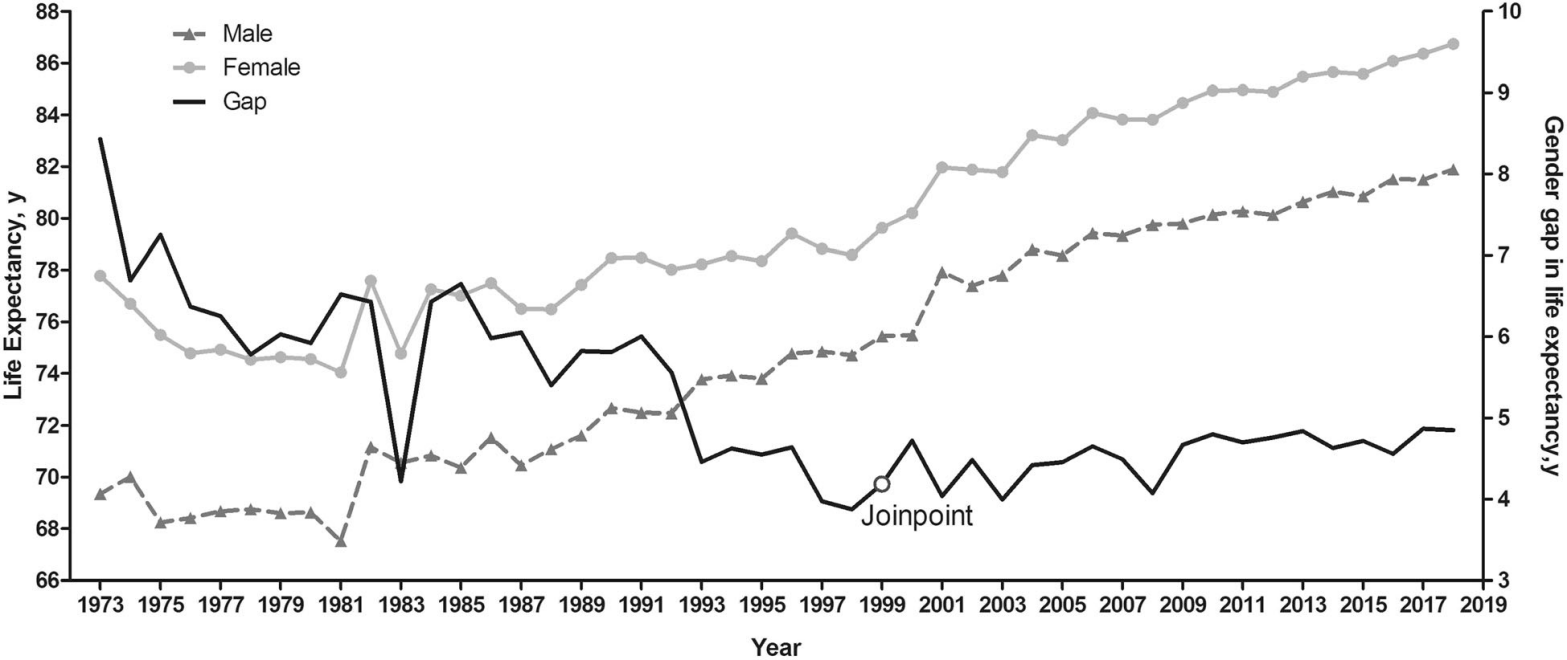
Life expectancy and its gender gap in Shanghai, 1973 to 2018. LE for both genders increased steadily after a short period of decrease and recovery during the 45 years

Figure 1 shows that male LE increased by 12.6 years, from 69.3 to 81.9, and female LE increased by 8.9 years, from 77.8 to 86.7, between 1973 and 2018 in Shanghai. The GGLE decreased from 8.4 years in 1973 to 4.8 years in 2018. Change of GGLE could be divided into two phases, the descent (1973 to 1999) saw a dramatic drop in GGLE from 8.4 to 4.2 (slope is − 0.11 with 95% CI (− 0.14, − 0.08)); the plateau (1999 to 2018) where GGLE fluctuated between 4.0 and 4.9 years. Joinpoint regression details can be referred to the Additional file 1: Table A2&A3.

### Age-specific contributions to GGLE at different phases

**Fig. 2:**
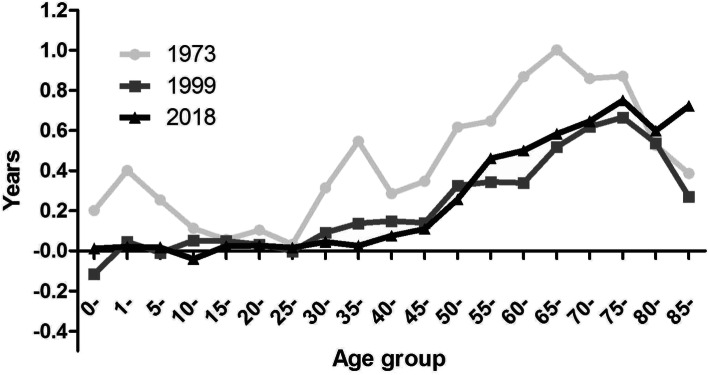
Age-specific contributions to GGLE in Shanghai in 1973, 1999 and 2018. Note: values above zero indicate males’ mortality rate is higher than females’ at specific age, vice versa

Figure 2 shows the age-specific contributions to GGLE in Shanghai in 1973, 1999, and 2018. Male mortality rates from all age groups were higher than female in 1973, primarily for age group 0–9 and above 30 years. Gender differences in the age-specific mortality rates in both 1999 (2.42‰) and 2018 (1.46‰) were significantly lower compared to 1973 (4.75‰). The reduced age-specific mortality rates concentrated in a narrower age range over time. Gender difference of the age-specific mortality rates mainly started from age 30 years in 1999, while excess male mortality mainly started from age 40 years in 2018. The age-specific contributions to GGLE were similar in 1999 and 2018, except for a wider GGLE at the advanced age, mainly from age over 55 years, and more pronounced at age above 85 years.

### Cause-specific contributions to GGLE

**Fig. 3:**
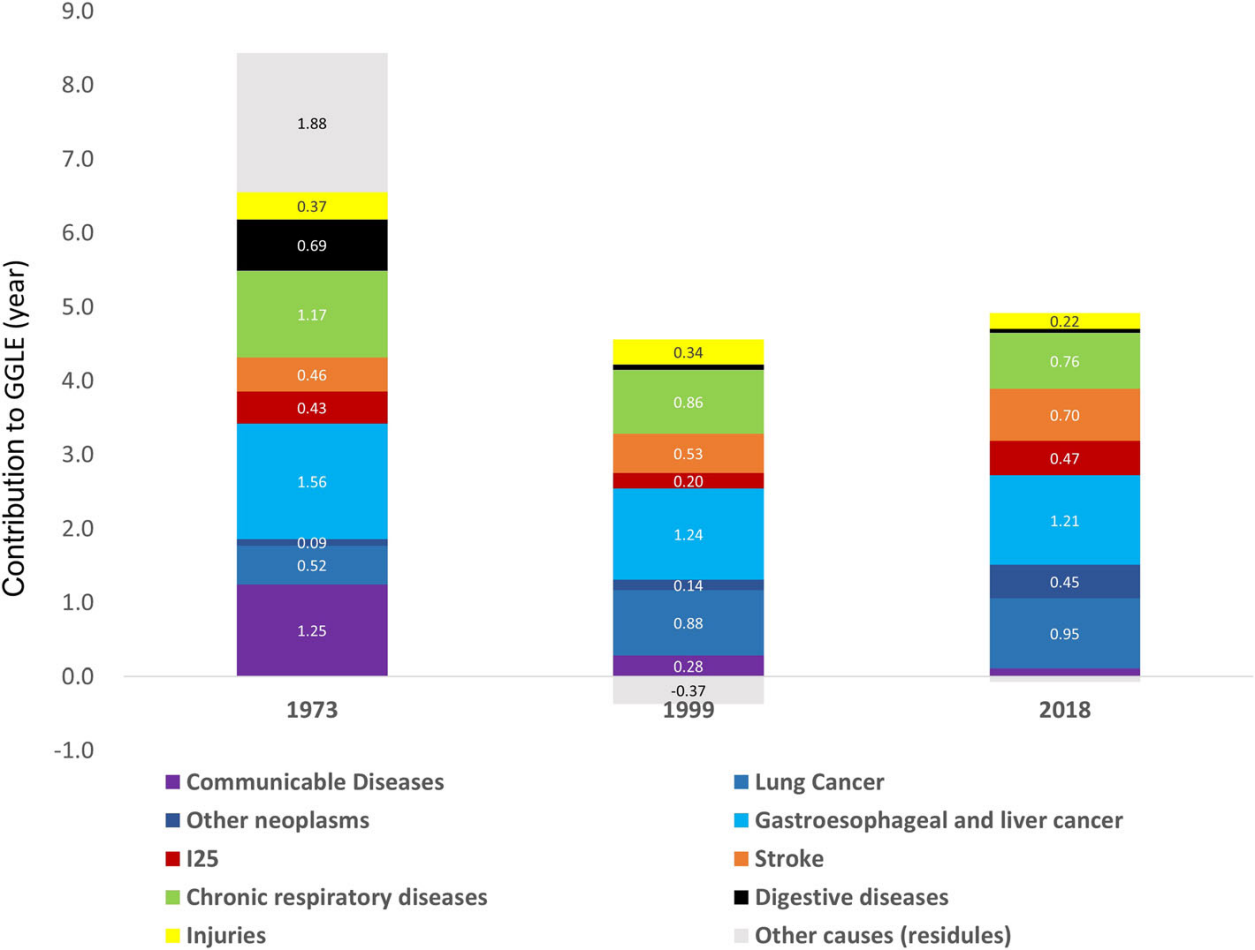
Cause-specific contributions to GGLE in Shanghai in 1973, 1999 and 2018. Values above zero indicate higher mortality in male. The sum of the positive and negative values results in GGLE of 8.4 years in 1973, 4.2 years in 1999 and 4.8 years in 2018

We divided the change of GGLE between 1973 and 2018 into two phases. Age-standardized all-cause mortality decreased by 29.8% in males and 12.9% in females during 1973 and 1999, while by 46.4 and 49.8% during 1999 and 2018. Table 1 shows the age-standardized mortalities of major cause of death for both genders. If male cause-specific mortality is higher than females, we regard this a positive contributor to GGLE, and vice versa. Figure 3 showed cause-specific contributions to GGLE in Shanghai in 1973, 1999 and 2018. All causes of death concerned in our study made positive contribution to GGLE in 1973. Among them, gastroesophageal and liver cancer, communicable diseases, chronic respiratory diseases, and digestive diseases made over half of the total contributions.

**Table 1:** Age-standardized mortality rates for main causes of death in Shanghai, China (1973, 1999, and 2018)

Cause of Death	Male						Female					
	1973		1999		2018		1973		1999		2018	
	Mortality	%	Mortality	%	Mortality	%	Mortality	%	Mortality	%	Mortality	%
All causes	1010.95	100	709.32	100	380.08	100	536.32	100	466.85	100	234.32	100
Communicable, maternal, neonatal, and nutritional diseases	85.57	9.26	31.60	4.04	6.76	1.45	26.49	5.08	22.50	3.60	3.72	1.27
1. Communicable diseases	83.73	9.02	27.96	3.92	5.45	1.37	24.05	0.00	15.68	3.34	2.52	1.15
2. Neonatal disorders	1.84	0.25	3.42	0.10	1.07	0.07	2.41	0.33	6.30	0.20	1.28	0.07
Non-communicable diseases	611.88	62.94	609.11	86.96	343.4	92.05	324.19	62.44	388.27	84.98	208.50	90.06
1. Neoplasms	184.11	21.19	220.69	34.58	138.04	36.01	80.96	15.84	103.65	21.95	73.17	25.22
I. Tracheal, bronchus, and lung cancer	36.01	4.32	67.73	10.87	43.23	11.55	9.67	1.91	18.75	4.10	14.04	5.04
II. Stomach cancer	43.96	4.82	33.29	5.29	16.30	4.32	19.03	3.83	13.49	2.99	7.20	2.44
III. Liver cancer	38.12	4.20	33.01	5.34	14.65	3.60	14.70	2.90	10.05	2.12	5.34	1.95
IV. Esophageal cancer	34.71	3.77	17.11	2.56	5.85	1.56	8.41	1.65	5.33	1.19	1.11	0.51
V. Other neoplasms	29.73	3.83	69.57	10.51	58.01	14.98	29.16	5.54	56.03	11.55	45.48	15.28
2. Cardiovascular diseases	201.44	19.89	218.07	29.99	126.62	34.98	141.56	27.13	174.34	39.21	87.24	44.38
I. Ischemic heart disease	107.44	10.75	62.89	8.36	59.92	16.89	80.47	15.25	49.22	11.08	44.71	24.04
II. Stroke	85.34	8.09	145.88	20.29	59.27	16.23	53.67	10.43	113.91	25.70	38.22	18.60
3. Chronic respiratory diseases	128.64	11.37	109.96	14.24	32.55	9.48	44.41	8.51	49.63	11.16	11.47	5.89
4. Digestive diseases	65.58	6.73	19.01	2.77	9.81	2.53	30.01	5.68	15.62	3.42	7.83	3.54
5. Diabetes, endocrine, blood, and immune disorders	1.32	1.17	13.86	3.06	16.40	6.40	3.04	1.85	17.79	5.12	12.89	1.27
6. Kidney diseases	7.86	0.99	7.04	0.93	7.77	2.07	6.33	1.19	5.13	1.08	5.14	2.05
Injuries	29.64	3.77	43.84	6.17	23.38	4.74	15.98	3.17	32.51	6.24	16.56	5.50

Note: Age-standardized death rate per 100 000 people and its corresponding percentage of total mortality

As shown in Fig. 3, in 1999, gastroesophageal and liver cancer, lung cancer, and chronic respiratory diseases were the leading contributors to GGLE, accounting for over 70% of the total contribution. Though gastroesophageal and liver cancer and chronic respiratory diseases remained the leading contributors, the contribution decreased. However, lung cancer made a greater contribution of 0.88 years to GGLE in 1999, compared with 0.52 years in 1973, with an excess of 0.36 years. In addition, communicable diseases fell out of the top three to make positive contribution to GGLE.

Gastroesophageal and liver cancer, lung cancer and chronic respiratory diseases remained the leading contributors to GGLE in 2018. Lung cancer itself made 0.95 years of contribution, while gastroesophageal and liver cancer in total made 1.21 years of contribution. It is worth noting, besides lung cancer, the contributions of cerebrovascular diseases and other neoplasms (except for gastroesophageal, liver and lung cancer) to GGLE were increasing during past 45 years. Cerebrovascular diseases contributed to GGLE from 0.46 years in 1973 to 0.70 years in 2018, just second to chronic respiratory diseases. Neoplasms except for gastroesophageal, liver and lung cancer increased the contribution from 0.09 years in 1973 to 0.45 years in 2018.

### Age- and cause-specific contributions to GGLE

**Fig. 4:**
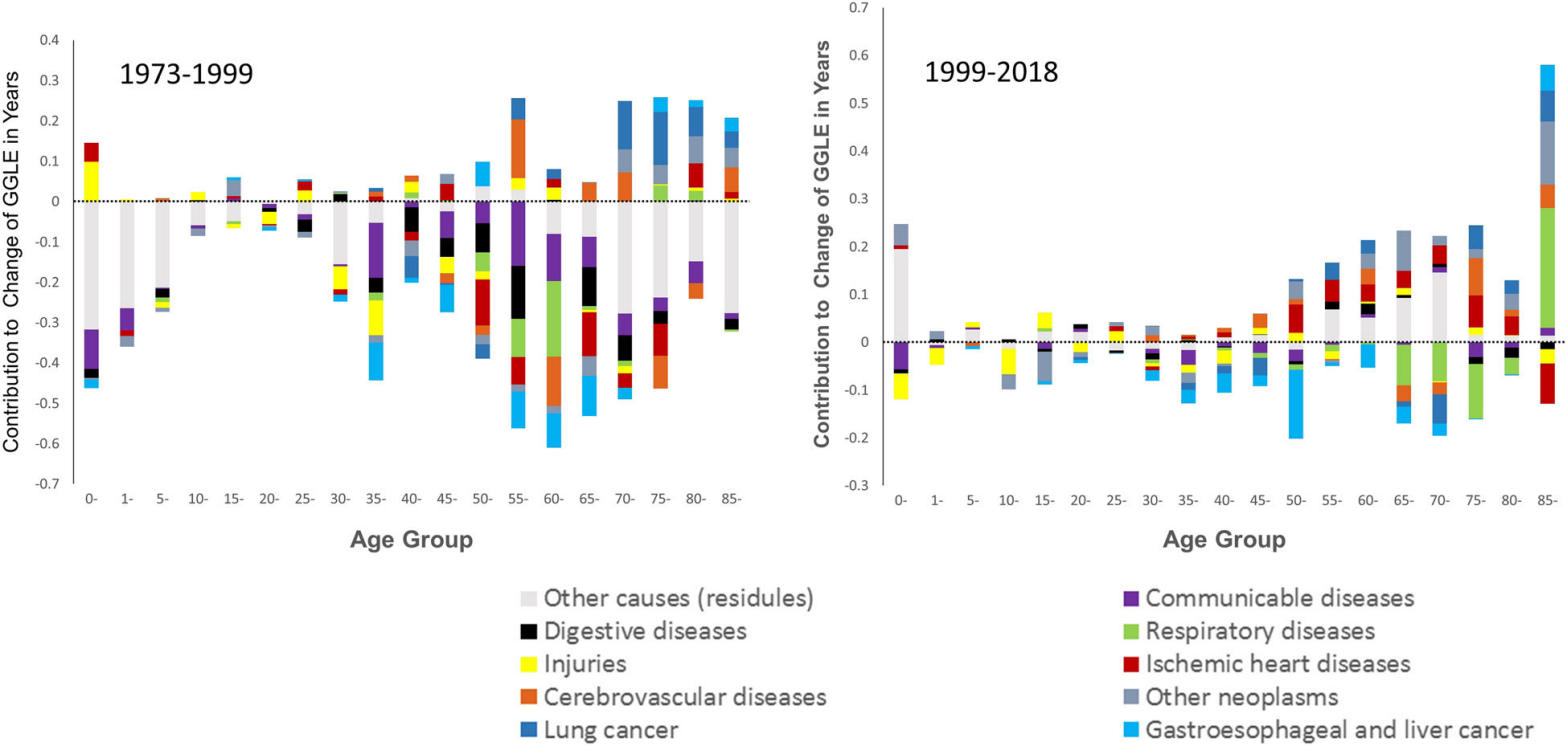
Age- and cause-specific contributions to the change of GGLE in Shanghai during 1973–1999 and 1999–2018. Positive values indicate a widening effect on GGLE by cause of death at corresponding age group, while negative values indicate a narrowing effect

Figure 4 shows the contribution of age and cause on GGLE. As shown, the age- and cause-specific contribution to GGLE concentrated more below the x-axis in the descent phase (1973–1999). Narrowing effect of GGLE mainly attributed to communicable, digestive diseases, liver cancer, and other causes of death at age 0–9 years; and communicable, digestive, chronic respiratory diseases, gastroesophageal, and liver cancer and other causes of death at age over 30 years. Meanwhile, lung cancer and other neoplasms widened GGLE, mainly at age over 70 years.

In the plateau phase (1999–2018), the contributions relatively balanced on both sides of x-axis. Nevertheless, it is worth noting that a widening GGLE attributed to cardiovascular diseases (ischemic heart diseases and cerebrovascular diseases), lung cancer, other neoplasms, and other causes of death (diabetes, endocrine, blood, and immune disorders, kidney diseases) at age over 55 years.

We compared the change of GGLE between 1973 and 1999 and 1999–2018. We found chronic respiratory diseases, neoplasms, and cerebrovascular diseases at the age group above 85 years made the biggest contribution to widen GGLE. Furthermore, neoplasms other than lung, gastroesophageal, and liver cancer widened GGLE at a broader age group. They used to widen GGLE mainly from age above 70 years at descent phase, but from age 50 years at the plateau phase.

## Discussion

Using data across 45 years (1973 to 2018), we studied changes in GGLE across time and contribution by age group and cause of death on changes in GGLE in Shanghai, China. We found two phases in the shift in GGLE. First, GGLE decreased by 4.2 years from 1973 to 1999, the descent phase, and then fluctuated around 4.5 years from 1999 to 2018, the plateau phase. Age-specific contribution to the change in GGLE concentrates in a narrow and more advanced age group (85 years and above). Non-communicable diseases (NCDs) such as cancer, cardiovascular, chronic respiratory diseases, and diabetes are major contributors to the changes in GGLE.

Communicable diseases used to be a major contributor to narrow GGLE in the descent phase (1973 to 1999). Nevertheless, its role diminished in the plateau phase (1999 to 2018). Shanghai suffered from hepatitis B, schistosomiasis, and tuberculosis during the descent phase [26]. Since the implementation of schistosomiasis control programs [27], hepatitis B vaccinations [28], modern tuberculosis control program and infectious disease surveillance [29], communicable diseases have been greatly reduced. This is a remarkable achievement in public health.

In China and globally, the achievement in communicable diseases corroborates an epidemiological shift to NCDs during the second half of the last century. NCDs accounted for an increasing proportion of total deaths, for about 60% of total deaths in 1973 and over 90% in 2018. Our study finds that gender differences in mortality due to NCDs are key to reduce GGLE in Shanghai, China. Male mortality of NCDs decreased by 43.9% and female by 35.7% over the past 45 years. NCDs like gastroesophageal and liver cancer, chronic respiratory and digestive diseases were the major contributors to narrow GGLE. While NCDs like lung cancer, other neoplasms, cardiovascular diseases, and diabetes were the major contributors to widen GGLE in our study.

Cancer is the largest contributor to GGLE in Shanghai. However, the effect of cancers on GGLE vary by cancer site. Gastroesophageal and liver cancer in total make a positive contribution to GGLE, but the contribution declines. Lung cancer and other neoplasms (except for the above-mentioned three cancers), however, continue to widen GGLE. Steady declines in mortality rates of gastroesophageal and liver cancers are potentially attributed from the overall achievement in public health [30], including HBV-vaccination, sanitation campaign (centralized drinking water supply system), improved treatment of parasitic liver fluke infections and healthy lifestyle changes (increased fresh fruits and vegetables, limited alcohol consumption, decreased salt-preservative/aflatoxin-contamination foods) [31–35].

Lung cancer has surpassed stomach cancer and become the leading cause of cancer death in Shanghai in recent years. The prevalence of smoking is much higher in male than female [36]. Lung cancer is one of the most preventable cancers [37]. Experience from the developed countries shows a marked decrease in smoking rates and lung cancer occurrence after a comprehensive tobacco control program [38]. Tobacco control can also reduce the risk of cardiovascular diseases [39]. Hence, Shanghai should take swift action to promote persistent efforts on tobacco control and attenuate the excess burden of smoking-related diseases experienced in developed countries, to promote the narrowing of GGLE.

Importantly, besides cancer, we also find a widening effect of GGLE by other NCDs like cardiovascular and endocrine diseases (diabetes). Overweight or obesity, sedentary lifestyle, hypertension, and hyperlipidemia are the main risk factors for cardiovascular and endocrine diseases (diabetes), which are more prevalent in males than in female [40–43]. Several exemplary policy interventions exists to prevent NCDs, such as promoting preventing health care through lifestyle behavioral changes and incorporate prevention into existing health care policy [44]. Another potential strategy is workplace health promotion to promote physical activity [42]. Further, we find an age-specific contribution to GGLE concentrate in a narrower age range as the decline of gains in LE, which is similar to findings in other high-LE countries [45, 46]. Promotion of physical activity, particularly among adults and the elderly seems relevant to our findings that suggest special attention to males at an advanced age for further improving LE and reducing GGLE.

The trend pattern and its main contributor to GGLE in Shanghai varied compared with other high-income countries or regions. In some western developed countries, GGLE is constantly narrowing [6, 8, 9, 45, 47], and cardiovascular diseases made the largest contribution to narrow GGLE [6, 9]. While in some eastern countries like Japan, GGLE is widening and cardiovascular diseases is an important contributor to the widening [9, 48]. We, however, find GGLE fluctuated between 4.0 and 4.9 in recent years, and cancer is the major driver to widen GGLE throughout the 45-year study period. It remains to see whether Shanghai’s steady GGLE in recent years will gradually narrow as in the western countries or expand as in China’s Asian neighbors.

Our study has several limitations. First, since the earliest data we have started from the year 1973, we do not know the trend of GGLE before. Many high-income countries found that GGLE experienced an expansion period and then declined through over 50 or more years of data. Limited by our data, we cannot make a comparison in this respect. However, given that we update the long-term trend study to the year 2018, guidance for future disease prevention and control to increase LE and narrow GGLE or at least not to expand is of greater significance. Second, we constrain the study only to major causes of death without breaking down the disease groups into more detailed causes (except for cardiovascular diseases and some major neoplasm). NCDs accounted for over 90% of total death in Shanghai in 2018, and cardiovascular diseases and neoplasms alone account for over 70% of total deaths. Therefore, we believe that no breakdown of disease groups such as injuries into more detailed causes has little impact on this study.

Third, our study only contains the information of local residents without that of flowing population and only analyzes GGLE by decomposition of age- and cause-specific contributions. Future research is expected to dive deeper into specific causes of death based on more detailed socio-economic data such as educational background and annual income with a comparison of different population.

Fourth, the 45-year-span is divided into two periods based on joinpoint regression result. Excluding data point with big fluctuation like 1983 does not affect the conclusion that GGLE in Shanghai has entered another phase in the late 1990s. Considering the decompositions of GGLE can provide meticulous change between years, we will also analyze age and cause-specific contributions according to some special time nodes for the development of Shanghai in the future study. Lastly, like other long-term trend studies, our data also faces data quality differences in different periods. The percentage of garbage code dropped greatly from above 40% in 1973 to 6.5% in 2018, which reached an excellent quality globally. We listed age-specific percentage of garbage codes in the Additional file 1: Figure A1.

NCDs are the biggest cause of death worldwide, and could have largely been prevented. Our findings showed that NCDs like cancer, cardiovascular, chronic respiratory, and diabetes were leading causes of death in Shanghai, and their proportions of total death have reached above 90%, efforts on prevention and control NCDs are imminent. China has implemented “Healthy China 2030” program, and one of its goals is to prevent and control four major NCDs (cardiovascular diseases, cancer, chronic respiratory diseases, and diabetes) and increase the national LE to 79 years by 2030. The Healthy China 2030 aims to ensure that everyone enjoys a full cycle and all-round health and promote health equity [49]. This also includes health equity between male and female. More specifically, as nations develop, the longitudinal pattern of declining GGLE turn around, and men start to catch up as evident in many high and middle-income countries.

Shanghai is the province with the highest LE in mainland China. The public health challenge facing Shanghai today, is also the challenge faced by other regions in China now and in the future (Additional file 1: Table A4). GGLE in Shanghai continues to persist in recent decades despite the ever-increasing LE. Our findings provide evidence for policymakers and health professionals in China and other countries with rapid gains in LE to formulate future strategies for medical resource allocation, system improvement, and disease management to address or prevent GGLE.

## Conclusions

NCDs have made GGLE enter a plateau phase from a descent phase in Shanghai China. Public efforts to reduce excess mortalities for male NCDs, cancers, cardiovascular diseases, chronic respiratory diseases, and diabetes in particular and health policies focused on the middle-aged and elderly population might further narrow GGLE and ensure improvement in health and health equity in Shanghai China.

## Supplementary information

**Additional file 1: Table S1.** List of causes of death focused in our study and the corresponding ICD-9 & ICD-10 codes. **Table S2.** Test for number of joinpoints. **Table S3.** Estimated regression coefficients for 1 joinpoint model. **Table S4.** Top 10 causes of death of China and Shanghai in 2017. **Figure S1.** Age-specific percentage of garbage codes in 1973, 1999 and 2018 in Shanghai.

## Data Availability

The datasets used and/or analysed during the current study available from the corresponding author on reasonable request with permission of Shanghai Pudong New area Center for Disease Control and Prevention.

## Abbreviations

*LE*: Life expectancy at birth
*GGLE*: Gender gap in life expectancy at birth
*NCD*: Non-communicable diseases
