# Supplementary material for: Non-communicable diseases are key to further narrow gender gap in life expectancy in Shanghai, China
Source: BMC Public Health. 2020 Jun 3;20:839. doi: 10.1186/s12889-020-08932-x (PMC7268263; doi:10.1186/s12889-020-08932-x)
Supplement: Supplementary file 1 — Additional file 1: Table S1. List of causes of death focused in our study and the corresponding ICD-9 & ICD-10 codes. Table S2. Test for number of joinpoints. Table S3. Estimated regression coefficients for 1 joinpoint model. Table S4. Top 10 causes of death of China and Shanghai in 2017. Figure S1. Age-specific percentage of garbage codes in 1973, 1999 and 2018 in Shanghai. [file 12889_2020_8932_MOESM1_ESM.docx]

**Data Supplement - Web appendix for**

**“**Non-communicable diseases are key to further narrow gender gap in life expectancy in Shanghai, China**”**

| **Table of Contents** | **Page** |
| --- | --- |
| **Table A1** List of causes of death focused in our study and the corresponding ICD-9 & ICD-10 codes | **1** |
| **Table A2** Test for number of joinpoints | **3** |
| **Table A3** Estimated regression coefficients for 1 joinpoint model | **3** |
| **Table A4** Top 10 causes of death of China and Shanghai in 2017 | **4** |
| **Figure A1** Age-specific percentage of garbage codes in 1973, 1999 and 2018 in Shanghai | **5** |

**Table A1** List of causes of death focused in our study and the corresponding ICD-9 & ICD-10 codes

| Cause | ICD-10 | ICD-9 |
| --- | --- | --- |
| Communicable diseases | A00-A00.9, A01.0-A14, A15-A28.9, A32-A39.9, A48.1-A48.2, A48.4-A48.5, A50-A58, A60-A60.9, A63-A63.8, A65-A65.0, A68-A70, A74, A74.8-A75.9, A77-A96.9, A98-A98.8, B00-B06.9, B10-B10.8, B15-B17.9, B19-B27.9, B29.4, B33-B33.1, B33.3-B33.8, B47-B48.8, B50-B53.8, B55.0, B56-B57.5, B60-B60.8, B63, B65-B67.9, B69-B72.0, B74.3-B75, B77-B77.9, B83-B83.8, B90-B91, B94.1-B94.2, B95-B95.5, B97.4-B97.6, D50.1-D50.8, D51-D52.0, D52.8-D53.9, E00-E02, E40-E46.9, E51-E61.9, E63-E64.0, E64.2-E64.9, F07.1, G00.0-G00.8, G03-G03.8, G04-G05.8, G14-G14.6, G21.3, H70-H70.9, I00, I02, I02.9, I98.0-I98.1, J00-J02.8, J03-J03.8, J04-J04.2, J05-J05.1, J06.0-J06.8, J09-J15.8, J16-J16.9, J20-J21.9, J36-J36.0, K67.0-K67.8, K75.3, K76.3, K77.0, K93.0-K93.1, M03.1, M12.1, M49.0-M49.1, M73.0-M73.1, M89.6, N74.1, P23.0-P23.4, P35-P35.2, P35.9, P37-P37.2, P37.5-P37.9, R19.7, U04-U04.9, U06-U06.9, U82-U89, Z16-Z16.3 | 001-001.9, 002.0-029, 032-034.9, 036-036.3, 036.5-037.9, 040, 040.1-041.0, 042-066.9, 070-075.9, 078.3-078.7, 079-079.7, 080-083.9, 084.0-084.5, 084.7-084.9, 085.0, 086-088, 088.8-088.9, 090-101.6, 104-104.9, 120-124.9, 125.4-125.9, 127-127.1, 128-129.0, 136-136.2, 137-139.0, 244.2, 260-263.9, 265-269.9, 281.0-281.9, 320.0-320.8, 321-323.9, 381-383.9, 390-390.9, 392, 392.9, 425.6, 460-464.4, 464.8-464.9, 465.0-465.8, 466-469, 470.0, 475-475.9, 476.9, 480-482.8, 483.0-483.9, 484.0-484.7, 487-489, 716.0, 730.4-730.6, 771.1-771.3, V09-V09.9 |
| Non-communicable diseases | A46-A46.0, A66-A67.9, B18-B18.9, B33.2, B86, C00-C13.9, C15-C25.9, C30-C34.9, C37-C38.8, C40-C41.9, C43-C45.9, C47-C54.9, C56-C57.8, C58-C58.0, C60-C63.8, C64-C67.9, C68.0-C68.8, C69-C75.8, C81-C86.6, C88-C96.9, D00.1-D00.2, D01.0-D01.3, D02.0-D02.3, D03-D06.9, D07.0-D07.2, D07.4-D07.5, D09.0, D09.2-D09.3, D09.8, D10.0-D10.7, D11-D12.9, D13.0-D13.7, D14.0-D14.3, D15-D16.9, D22-D27.9, D28.0-D28.7, D29.0-D29.8, D30.0-D30.8, D31-D36, D36.1-D36.7, D37.1-D37.5, D38.0-D38.5, D39.1-D39.2, D39.8, D40.0-D40.8, D41.0-D41.8, D42-D43.9, D44.0-D44.8, D45-D47.9, D48.0-D48.6, D49.2-D49.4, D49.6, D52.1, D55-D58.9, D59.0-D59.3, D59.5-D59.6, D60-D61.9, D63.1, D64.0, D66-D67, D68.0-D69.8, D70-D75.8, D76-D78.8, D86-D86.9, D89-D89.3, E03-E07.1, E09-E11.9, E15.0, E16.0-E16.9, E20-E34.8, E36-E36.8, E65-E68, E70-E85.2, E88-E89.9, F00-F03.9, F10-F16.9, F18-F19.9, F24, F50.0-F50.5, G10-G13.8, G20-G20.9, G23-G26.0, G30-G31.9, G35-G37.9, G40-G41.9, G45-G46.8, G47.3, G61-G61.9, G70-G73.7, G90-G90.9, G93.7, G95-G95.9, G97-G97.9, H05.0-H05.1, I01-I01.9, I02.0, I05-I09.9, I11-I13.9, I20-I25.9, I27.1, I28-I28.8, I30-I31.1, I31.8-I37.8, I38-I41.9, I42.1-I42.8, I43-I43.9, I47-I48.9, I51.0-I51.4, I60-I63.9, I65-I66.9, I67.0-I67.3, I67.5-I67.7, I68.0-I68.2, I69.0-I69.3, I70.2-I70.8, I71-I73.9, I77-I89.9, I95.2-I95.3, I97-I98, I98.2, I98.9, J30-J35.9, J37-J39.9, J41-J46.9, J60-J63.8, J65-J68.9, J70-J70.9, J82, J84-J84.9, J91-J92.9, J95-J95.9, K20-K29.9, K31-K31.8, K35-K38.9, K40-K46.9, K50-K52.9, K55-K62.9, K63.5, K64-K64.9, K66.8, K67, K68-K68.9, K70-K70.3, K71.7, K74-K74.9, K75.1-K75.2, K75.4-K76.2, K76.4-K77, K77.8, K80-K83.9, K85-K86.9, K90-K91.9, K92.8, K93.8-K95.8, L00-L05.9, L08-L08.9, L10-L14.0, L51-L51.9, L88-L89.9, L93-L93.2, L97-L98.4, M00-M03.0, M03.2-M03.6, M05-M09.8, M30-M36.8, M40-M43.1, M65-M65.0, M71.0-M71.1, M72.5-M72.6, M80-M82.8, M86.3-M86.4, M87-M87.1, M88-M89.0, M89.5, M89.7-M89.9, N00-N08.8, N10-N12.9, N14-N16.8, N18-N18.9, N20-N23.0, N25-N28.1, N29-N32.0, N32.3-N32.4, N34-N34.3, N36-N36.9, N39-N39.2, N41-N41.9, N44-N44.0, N45-N45.9, N49-N49.9, N60-N60.9, N65-N65.1, N72-N72.0, N75-N77.8, N80-N81.9, N83-N83.9, N84.0-N84.1, N87-N87.9, N99-N99.9, P04.3-P04.4, P70.2, P96.0-P96.2, P96.5, Q00-Q07.9, Q10.4-Q18.9, Q20-Q28.9, Q30-Q36, Q37-Q45.9, Q50-Q87.8, Q89-Q89.8, Q90-Q93.9, Q95-Q99.8, R50.2, R78.0-R78.5, R95-R95.9, X45-X45.9, X65-X65.9, Y15-Y15.9 | 035-035.9, 036.4, 102-103.9, 133-133.6, 135-135.9, 136.6, 140-148.9, 150-158.9, 160-164.9, 170-175.9, 180-183.8, 184.0-184.4, 184.8, 185-186.9, 187.1-187.8, 188-188.9, 189.0-189.8, 190-194.8, 200-208.9, 209.0-209.1, 209.4-209.5, 210.0-210.9, 211.0-211.8, 212.0-212.8, 213-213.9, 217-220.9, 221.0-221.8, 222.0-222.8, 223.0-223.8, 224-228.9, 229.0, 229.8, 230.1-230.8, 231.0-231.2, 232-232.9, 233.0-233.2, 233.4-233.5, 233.7, 234.0-234.8, 235.0, 235.4, 235.6-235.8, 236.0-236.2, 236.4-236.5, 236.7, 237-237.3, 237.5-237.9, 238.0-238.9, 239.2-239.4, 239.6, 240-243.9, 244.0-244.1, 244.3-244.8, 245-246.9, 251-259.9, 270-273.9, 275-276, 277-277.2, 277.4-277.9, 278.0-278.8, 282-284.9, 286-286.5, 286.7-289.7, 290-292.9, 294.1-294.9, 303-303.9, 304.0-304.8, 305-305.9, 307.1, 327.2-327.8, 330-331.2, 331.5-332.0, 333-337.9, 340-341.9, 345-345.9, 349, 349.2-349.8, 353.6-353.9, 356-356.9, 357.0-357.1, 357.3-357.7, 358-359.9, 376.0-376.1, 391-391.9, 392.0, 393-398.9, 402-404.9, 410-414.9, 416.1, 417-417.9, 420-423, 423.1-423.9, 424.0-424.9, 425.0-425.3, 425.5, 425.7-425.8, 427-427.3, 427.6-427.8, 429.0, 430-435.9, 437.0-437.2, 437.4-437.8, 440.2, 440.4, 441-443.9, 446-457, 457.1-457.9, 459, 459.1-459.3, 470, 470.9-474.9, 476-476.1, 477-479, 491-493.9, 495-504.9, 506-506.9, 508-509, 515, 516-517.8, 518.6-518.7, 518.9, 519.0-519.4, 530-536.1, 536.4, 537-537.6, 537.8, 538-543.9, 550-553.6, 555-558.9, 560-560.3, 560.8-560.9, 562-562.1, 564-564.7, 565-566.9, 569.0-569.7, 571-571.9, 572.2-573.0, 573.4-577.9, 579-583.9, 585-585.9, 588-590.9, 592-593.8, 594-599.6, 599.8, 601-602.9, 604-604.9, 608.2, 610-610.9, 617-618.9, 620-620.9, 621.4-621.9, 622.1-622.7, 629-629.8, 680-689, 694-695.5, 707-707.9, 710-711.9, 714-714.3, 714.8-714.9, 730.1, 732-732.9, 733.0-733.1, 740-749.0, 749.2-758.9, 759.0-759.8, 760.7, 775.1-775.3, 779.4-779.5, 787.1, 788.0, 790.3, 798-798.0, E850, E860 |
| Cardiovascular diseases | B33.2, G45-G46.8, I01-I01.9, I02.0, I05-I09.9, I11-I11.9, I20-I25.9, I28-I28.8, I30-I31.1, I31.8-I37.8, I38-I41.9, I42.1-I42.8, I43-I43.9, I47-I48.9, I51.0-I51.4, I60-I63.9, I65-I66.9, I67.0-I67.3, I67.5-I67.6, I68.0-I68.2, I69.0-I69.3, I70.2-I70.8, I71-I73.9, I77-I83.9, I86-I89.0, I89.9, I98, K75.1 | 036.4, 391-391.9, 392.0, 393-398.9, 402-402.9, 410-414.9, 417-417.9, 420-423, 423.1-423.9, 424.0-424.9, 425.0-425.3, 425.5, 425.7-425.8, 427-427.3, 427.6-427.8, 429.0, 430-435.9, 437.0-437.2, 437.5-437.8, 440.2, 440.4, 441-443.9, 447-454.9, 456, 456.3-457, 457.1, 457.8-457.9, 459, 459.1-459.3 |
| Ischemic heart disease | I20-I25.9 | 410-414.9 |
| Cerebrovascular disease | G45-G46.8, I60-I63.9, I65-I66.9, I67.0-I67.3, I67.5-I67.6, I68.1-I68.2, I69.0-I69.3 | 430-435.9, 437.0-437.2, 437.5-437.8 |
| Chronic respiratory diseases | D86-D86.2, D86.9, G47.3, J30-J35.9, J37-J39.9, J41-J46.9, J60-J63.8, J65-J68.9, J70, J70.8-J70.9, J82, J84-J84.9, J91-J92.9 | 135-135.9, 136.6, 327.2-327.8, 470, 470.9-474.9, 476-476.1, 477-479, 491-493.9, 495-504.9, 506-506.9, 508-509, 515, 516-517.8, 518.6, 518.9, 519.1-519.4 |
| Digestive diseases | B18-B18.9, I84-I85.9, I98.2, K20-K29.9, K31-K31.8, K35-K38.9, K40-K42.9, K44-K46.9, K50-K52.9, K55-K62, K62.2-K62.6, K62.8-K62.9, K64-K64.9, K66.8, K67, K68-K68.9, K70-K70.3, K71.7, K74-K74.9, K75.2, K75.4-K76.2, K76.4-K77, K77.8, K80-K83.9, K85-K86.9, K90-K90.9, K92.8, K93.8, M09.1 | 455-455.9, 456.0-456.2, 530-536.1, 537-537.6, 537.8, 538, 540-543.9, 550-551.1, 551.3-552.1, 552.3-553.6, 555-558.9, 560-560.3, 560.8-560.9, 562-562.1, 564-564.1, 564.5-564.7, 565-566.9, 569.1-569.5, 569.7, 571-571.9, 572.2-573.0, 573.4-577.9, 579-579.2, 579.4-579.9, 787.1 |
| Diabetes and kidney diseases, endocrine, metabolic, blood, and immune disorders | D63.1, E10-E11.9, I12-I13.9, N00-N08.8, N15.0, N18-N18.9, P70.2, Q61-Q62.8,D52.1, D59.0, D59.2, D59.6, D66-D67, D68.0-D69.8, D70-D75.8, D76-D78.8, D86.8, D89-D89.3, E03-E07.1, E09-E09.9, E15.0, E16.0-E16.9, E20-E28.1, E28.3-E34.8, E36-E36.8, E65-E68, E70-E85.2, E88-E89.9, G24.0, G25.1, G25.4, G25.6-G25.7, G72.0, G93.7, G97-G97.9, I95.2-I95.3, I97-I97.9, I98.9, J70.0-J70.5, J95-J95.9, K43-K43.9, K62.7, K91-K91.9, K94-K95.8, M87.1, N14-N14.4, N65-N65.1, N99-N99.9, P96.2, P96.5, R50.2 | 403-404.9, 580-583.9, 585-585.9, 589-589.9, 753-753.3, 775.1, 240-243.9, 244.0-244.1, 244.3-244.8, 245-246.9, 251-256.3, 256.8-259.9, 270-273.9, 275-276, 277-277.2, 277.4-277.9, 278.0-278.8, 286-286.5, 286.7-289.7, 357.6, 518.7, 519.0, 536.4, 539-539.9, 551.2, 552.2, 564.2-564.4, 569.6, 579.3, 598.2, 775.3, 779.4-779.5 |
| Diabetes mellitus | E10-E10.1, E10.3-E11.1, E11.3-E11.9, P70.2 | 775.1 |
| Chronic kidney disease | D63.1, E10.2, E11.2, I12-I13.9, N02-N08.8, N15.0, N18-N18.9, Q61-Q62.8 | 403-404.9, 581-583.9, 585-585.9, 589-589.9, 753-753.3 |
| Neoplasms | C00-C13.9, C15-C25.9, C30-C34.9, C37-C38.8, C40-C41.9, C43-C45.9, C47-C54.9, C56-C57.8, C58-C58.0, C60-C63.8, C64-C67.9, C68.0-C68.8, C69-C75.8, C81-C86.6, C88-C96.9, D00.1-D00.2, D01.0-D01.3, D02.0-D02.3, D03-D06.9, D07.0-D07.2, D07.4-D07.5, D09.0, D09.2-D09.3, D09.8, D10.0-D10.7, D11-D12.9, D13.0-D13.7, D14.0-D14.3, D15-D16.9, D22-D24.9, D26.0-D27.9, D28.0-D28.1, D28.7, D29.0-D29.8, D30.0-D30.8, D31-D36, D36.1-D36.7, D37.1-D37.5, D38.0-D38.5, D39.1-D39.2, D39.8, D40.0-D40.8, D41.0-D41.8, D42-D43.9, D44.0-D44.8, D45-D47.9, D48.0-D48.6, D49.2-D49.4, D49.6, K62.0-K62.1, K63.5, N60-N60.9, N84.0-N84.1, N87-N87.9 | 140-148.9, 150-158.9, 160-164.9, 170-175.9, 180-183.8, 184.0-184.4, 184.8, 185-186.9, 187.1-187.8, 188-188.9, 189.0-189.8, 190-194.8, 200-208.9, 209.0-209.1, 209.4-209.5, 210.0-210.9, 211.0-211.8, 212.0-212.8, 213-213.9, 217-217.8, 219.0, 220-220.9, 221.0-221.8, 222.0-222.8, 223.0-223.8, 224-228.9, 229.0, 229.8, 230.1-230.8, 231.0-231.2, 232-232.9, 233.0-233.2, 233.4-233.5, 233.7, 234.0-234.8, 235.0, 235.4, 235.6-235.8, 236.1-236.2, 236.4-236.5, 236.7, 237-237.3, 237.5-237.9, 238.0-238.9, 239.2-239.4, 239.6, 569.0, 610-610.9, 622.1-622.2, 622.7 |
| Esophageal cancer | C15-C15.9, D00.1, D13.0 | 150-150.9, 211.0, 230.1 |
| Stomach cancer | C16-C16.9, D00.2, D13.1, D37.1 | 151-151.9, 211.1, 230.2 |
| Liver cancer | C22-C22.9, D13.4 | 155-155.9, 211.5 |
| Lung cancer | C33-C34.9, D02.1-D02.3, D14.2-D14.3, D38.1 | 162-162.9, 212.2-212.3, 231.1-231.2, 235.7 |
| Injuries | L55-L55.9, L56.3, L56.8-L56.9, L58-L58.9, U00-U03, V00-V86.9, V87.2-V87.3, V88.2-V88.3, V90-V98.8, W00-W46.2, W49-W62.9, W64-W70.9, W73-W75.9, W77-W81.9, W83-W94.9, W97.9, W99-X06.9, X08-X39.9, X46-X48.9, X50-X54.9, X57-X58.9, X60-X64.9, X66-Y08.9, Y35-Y84.9, Y87.0-Y87.1, Y88-Y88.3, Y89.0-Y89.1 | 349.0-349.1, 457.0, E800-E807, E830-E838, E840-E849, E856-E857, E861-E865, E867-E869, E870-E876, E878-E879, E880-E886, E888-E928, E930-E979, E990-E999 |

**Table A2** Test for number of joinpoints

| Test | Null hypothesis | Alternate hypothesis | *P* | Significance level |
| --- | --- | --- | --- | --- |
| 1 | 0 Joinpoints | 5 Joinpoints* | <0.001 | 0.0100 |
| 2 | 1 Joinpoint* | 5 Joinpoints | 0.088 | 0.0125 |
| 3 | 1 Joinpoint* | 4 Joinpoints | 0.054 | 0.0125 |
| 4 | 1 Joinpoint* | 3 Joinpoints | 0.022 | 0.0125 |
| 5 | 1 Joinpoint* | 2 Joinpoints | 0.226 | 0.0125 |

* Final Selected Model

1 Joinpoint model was selected and the trend of GGLE was divided into two phases, 1973-1999 and 1999-2018.

**Table A3** Estimated regression coefficients for 1 joinpoint model

| Parameter | Estimate | SE | *Z* |
| --- | --- | --- | --- |
| Intercept 1 | 226.48 | 27.07 | 8.365*** |
| Intercept 2 | -56.61 | 43.88 | -1.290 |
| Slope 1 | -0.11 | 0.01 | -8.152*** |
| Slope 2 | 0.03 | 0.02 | 1.394 |

* *P*-value<0.05; ** *P* -value<0.01; *** *P* -value<0.001

**Table A4** Top 10 causes of death of China and Shanghai in 2017

| Rank | China^a^ | |  | Shanghai^b^ | |
| --- | --- | --- | --- | --- | --- |
|  | Cause | % |  | Cause | % |
| 1 | Cardiovascular | 43.6 |  | Cardiovascular | 40.4 |
| 2 | Cancer | 26.1 |  | Cancer | 30.5 |
| 3 | Respiratory | 10.9 |  | Respiratory | 8.4 |
| 4 | External | 5.9 |  | Endocrine | 5.3 |
| 5 | Endocrine | 3.3 |  | External | 4.7 |
| 6 | Digestive | 2.4 |  | Digestive | 2.3 |
| 7 | Nervous | 1.3 |  | Nervous | 1.4 |
| 8 | Infectious | 1.0 |  | Infectious | 0.9 |
| 9 | Genitourinary | 1.1 |  | Mental | 1.1 |
| 10 | Mental | 0.4 |  | Genitourinary | 0.9 |

1. China Statistical Yearbook 2018; b. Shanghai Statistical Yearbook 2018

The leading causes of death for China and Shanghai were similar in rank and type based on CCD (Chinese Classification of Diseases). NCDs being the leading causes of death, cardiovascular and cancer in total account for 70% of total death for both China and Shanghai.


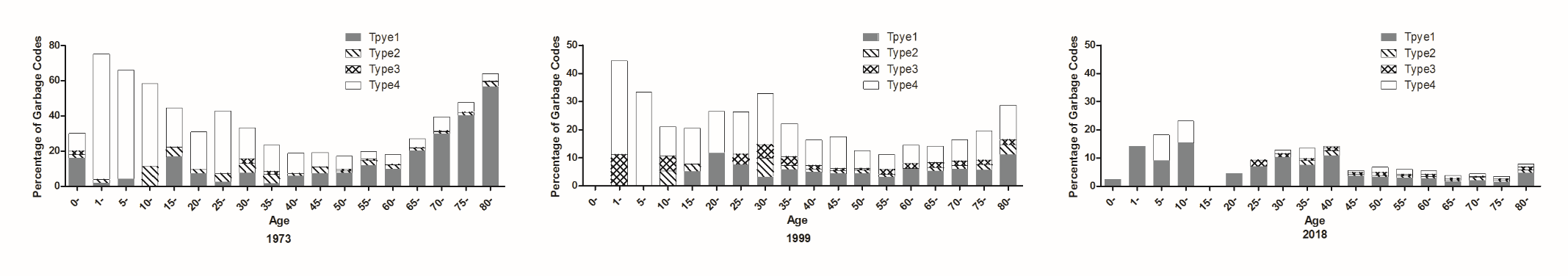


**Figure A1 Age-specific percentage of garbage codes in 1973, 1999 and 2018 in Shanghai.** According to Global Burden of Disease (GBD) study, garbage codes are classified into four categories: Type 1- causes that cannot or should not be considered as underlying causes of death. Type 2- intermediate causes of death such as heart failure, peritonitis and septicemia, etc al. Type 3- immediate causes of death that are the final steps in a disease pathway leading to death. Type 4- unspecified causes within a larger cause grouping.

The type-1 garbage codes are mainly sudden death and age-related physical debility aggregating in age over 40 years in 1973, while be cerebral palsy, developmental retardation and sudden death at home in 2018. Considering the cause of death concerned in our study, we hold that garbage codes, on the whole, have little influence on our major findings except that it might underestimate contribution of cardiovascular diseases to GGLE in historical data.
